# Vertebral fracture and spinal cord compression after removal of pedicle screws in PD patients: case report and literature review

**DOI:** 10.3389/fsurg.2025.1690927

**Published:** 2025-12-03

**Authors:** Jingchao Wen, Yingfeng Su, Jiandong Guo, Shunwu Fan, Xiangqian Fang, Junxin Chen, Wenbin Xu

**Affiliations:** 1 Orthopedics Department, School of Medicine, Sir Run Run Shaw Hospital, Zhejiang University, Hangzhou, China; 2 Department of Orthopaedics, Hangzhou Ninth People’s Hospital, Hangzhou, China

**Keywords:** pedicle screw, failed back surgery syndrome, screw removal, case report, vertebral fracture, spinal cord compression, spinal surgery

## Abstract

**Introduction:**

Pedicle screws are used in spinal surgery and have yielded favorable postoperative outcomes. Despite this, the optimal timing for their removal remains controversial. This study presents a case report of a patient with Parkinson's disease who experienced rapid vertebral fracture and spinal cord compression after pedicle screw removal. We outlined the patient's management, and follow up and discuss consideration for screw removal.

**Case presentation:**

A 58-year-old woman with an 8-year history of Parkinson's disease underwent lumbar surgery (L4-L5 oblique lumbar interbody fusion, L5-S1 transverse lumbar interbody fusion, and T12-L4 posterolateral lumbar fusion), including pedicle screw implantation at a local hospital 3 years prior for degenerative lumbar spondylolisthesis and lumbar spinal stenosis. She presented with lower back pain and bilateral lower limb numbness for 7 months. The patient was diagnosed with failed back surgery syndrome and underwent posterior fixation removal, interbody fusion cage removal, decompression, fusion with bone grafting, and new internal fixation (L4-S2). However, 3 months postoperatively, the lower back pain recurred and worsened by the fourth month, accompanied by lower limb weakness. Subsequently, the patient was diagnosed with L2 and L3 vertebral fractures and spinal cord injury.

**Results:**

With appropriate treatment including total L2 laminectomy with decompression, intertransverse bone grafting, L2 vertebroplasty, pedicle screw-rod fixation (T11-S2), and T11 screw tract augmentation, the patient exhibited a satisfactory prognosis at the 2-year follow-up.

## 1 Introduction

A pedicle screw is an orthopedic surgical device designed to provide robust spinal fixation via insertion through the pedicle. The concept of posterior spinal instrumentation dates back to the use of spinous processes, with Dr. Hadra applying it to treat Pott's disease in 1891. The modern pedicle screw prototype was introduced by Professor Boucher, who successfully performed lumbosacral fusion surgery in 1959 by inserting pedicle screws through the vertebral pedicles into the vertebral bodies. Roy and Camille developed the first comprehensive pedicle screw fixation system in 1963 (1, 2). Professor Tian-Si Tang from Suzhou Medical University, China achieved the earliest domestic application of this technology in 1989 (3). With rapid advances in spinal surgery, pedicle screw systems have evolved through continuous innovation, incorporating various configurations such as fixed-angle screws, polyaxial screws, and cement-augmented screw systems. These systems are the preferred method for posterior spinal fixation owing to their multiple advantages including ease of screw placement, superior mechanical stability, broad clinical applicability, and the ability to facilitate spinal manipulation such as reduction, distraction, traction, and compression intraoperatively. Additionally, their compatibility with diverse surgical requirements has established them as the preferred instrumentation for contemporary spinal stabilization procedures.

Although pedicle screws are covered by paraspinal musculature, they may remain palpable in emaciated patients or during certain positional maneuvers, potentially causing discomfort. Some patients experience persistent postoperative low back pain even after excluding common etiologies such as nerve root compression, pseudoarthrosis, or surgical site infection (4, 5), prompting the clinical suspicion of screw-related pain. Consequently, whether pedicle screws should be removed after healing is a question that frequently arises during patient consultations, occasionally posing a dilemma for clinicians. In cases of spinal fractures treated solely with pedicle screw fixation, hardware removal is generally recommended to restore partial segmental mobility. Revision procedures for adjacent segment disease and proximal junctional kyphosis have become more prevalent with rising life expectancies and increasing spinal surgery volumes. In revision scenarios involving previously fused segments, the existing rod-screw constructs are typically removed, with selective screw replacement used as required for adjacent-level stabilization. This clinical context raises two critical questions. 1) Should routine removal of thoracolumbar pedicle screws be standard practice post-fracture stabilization? 2) In revision surgeries, should pre-existing pedicle screw systems be completly removed or partially replaced?

Here, we report a case involving a 58-year-old female with an 8-year history of Parkinson's disease (PD) who underwent lumbar 4–5 (L4-L5) oblique lumbar interbody fusion (OLIF) surgery, but subsequently developed failed back surgery syndrome. Successive L2 and L3 vertebral fractures with spinal cord injury rapidly developed following surgical treatment for the failed back surgery syndrome. Despite this, the patient showed notable recovery and demonstrated potential for successful rehabilitation. This case report reviews the specific surgical procedures involved and provided a comprehensive literature review to guide clinical decision-making on the removal of internal fixation devices, including whether and when it is required.

## 2 Case presentation

### 2.1 Clinical information

A 58-year-old woman with an 8-year history of PD underwent L4-L5 OLIF, lumbar vertebrae (L) 5—sacral vertebrae (S) 1 transforaminal lumbar interbody fusion and thoracic vertebrae (T)—L4 posterolateral fusion surgery at a local hospital 3 years prior, owing to degenerative lumbar scoliosis and lumbar spinal stenosis. Approximately 7 months before admission, she developed lower back pain along with pain and numbness in both lower extremities. The numbness was more pronounced in the left leg, and the pain worsened while walking. Her PD was well controlled with a combination regimen comprising of carbidopa/levodopa compound preparation tablets, pramipexole hydrochloride, entacapone, amantadine hydrochloride, and levodopa/benserazide hydrochloride tablets. Her balance could be maintained though both lower extremities are affected. According to her clinical manifestations, she was evaluated as stage 2 according to Hoehn-Yahr stage. The rest of her family are in good health conditions.

### 2.2 Examination

**Figure 1:**
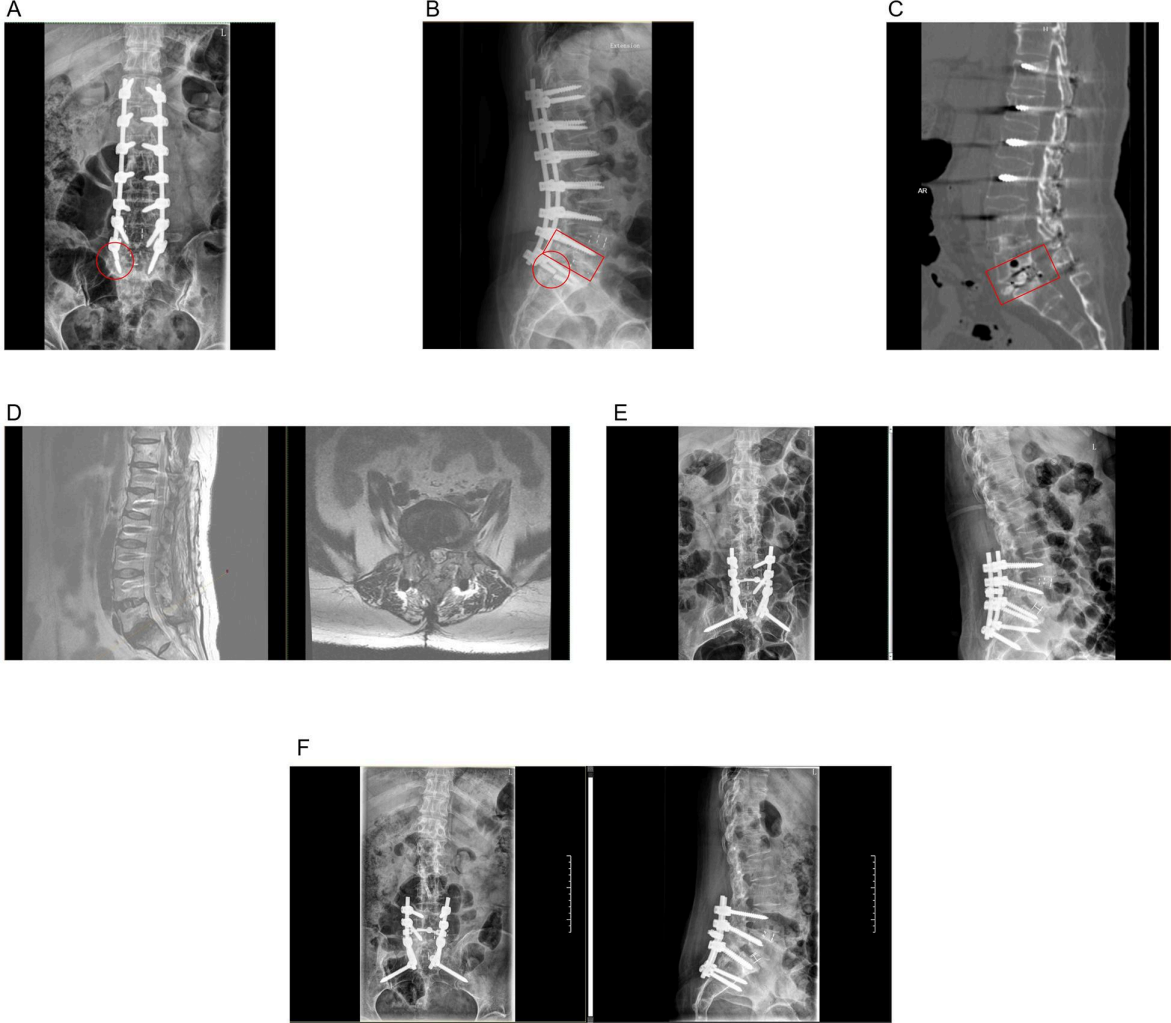
**(A,B)** Preoperative anteroposterior and lateral radiographs showing the broken screw (red circle), and the non-union of the cage (red rectangle). **(C)** Computed tomography (CT) image showing an unfused cage (red rectangle). **(D)** Saggital and axial magnetic resonance images (MRI) images showing no spinal cord injury. **(E)** Postsurgical posterolateral radiograph. **(F)** Posterolateral radiograph during 1.5 months’ follow-up.

Surgical incision scars were observed in the lower back region during physical examination. Additionally, superficial sensation was diminished in the left calf and posterior thigh. Sensation in the right lower extremity remained intact. Dorsiflexion strength of both halluces was graded as 4/5. The patient reported a pain score of 6 out of 10 on the visual analogue scale. Electromyography suggested possible bilateral neural damage at the L2-S1 level. Dual-energy x-ray absorption revealed a normal density (*T* = 0.4 in femur). Preoperative computed tomography revealed fusion failure at L5/S1 and broken screws at the S1 level. Magnetic resonance imaging showed no evidence of spinal cord injury (Figures 1A–D).

## 3 Diagnosis

### 3.1 Diagnostic assessment

The patient was diagnosed with fusion failure, which was accompanied by screw breakage. Upon assessing her previous surgical plan and prognosis, we assumed that long-segment fusion involving only partial posterolateral segments rather than intervertebral fusion lacks mechanical strength and a higher potency for proximal failure. Based on these findings, we developed a surgical plan comprising posterior L1-S1 fixation removal, L5-S1 interbody cage removal, decompression and fusion using bone grafting and new fusion cage placement, and L4-L5 inner fixation with S2-alar-iliac fixation. No intra-operative complications were observed. Postoperative radiographs are shown in Figure 1E. The patient ambulated with spinal bracing 3 days after surgery and demonstrated a good short-term prognosis at her scheduled follow-up after 1.5 months (Figure 1F).

**Figure 2:**
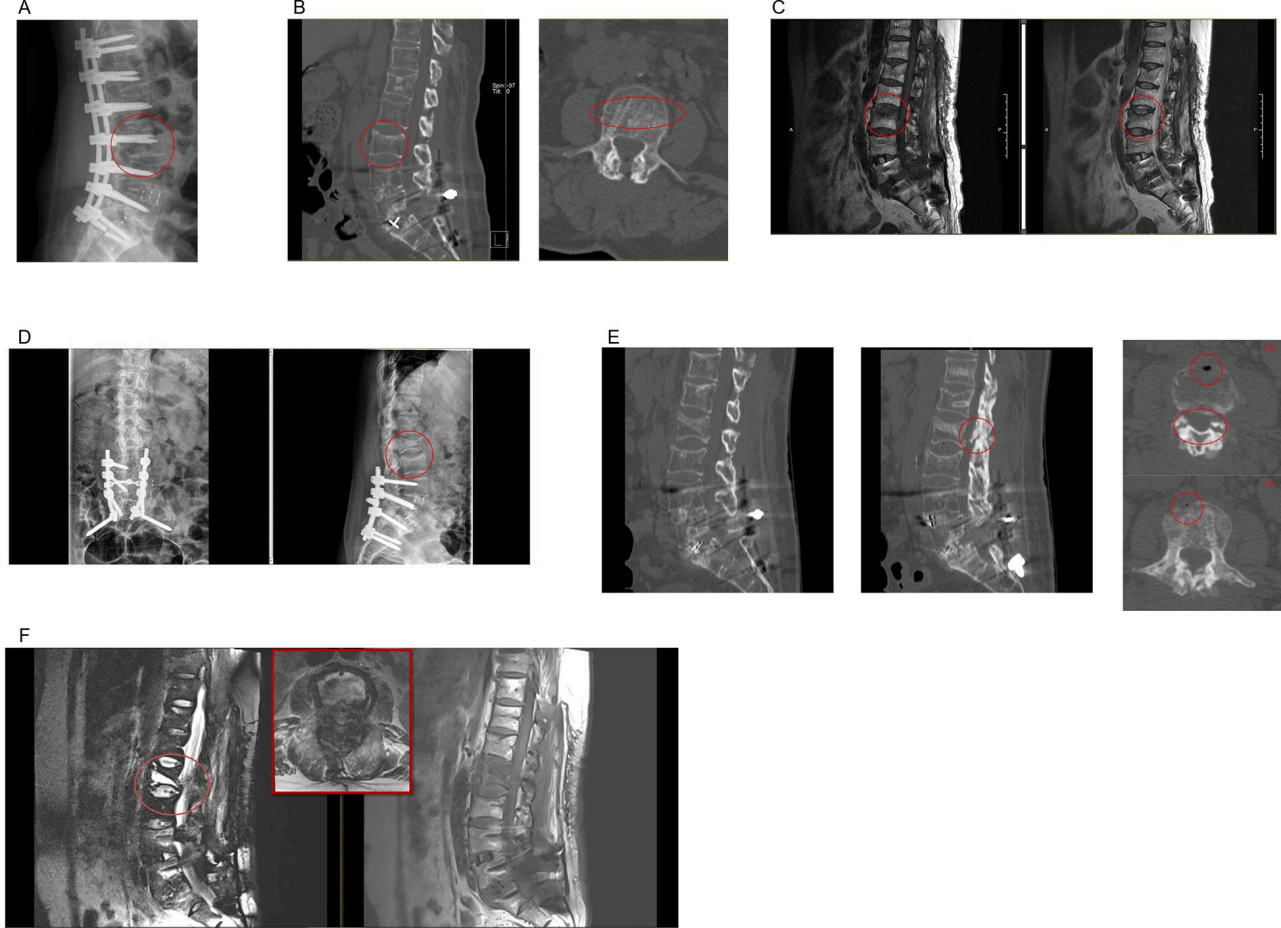
**(A,B)** Radiograph **(A)** and corresponding sagittal (left) and transverse (right) CT images **(B)** showing the same vertebrae marked with a red circle. The fracture line is marked with a red oval. **(C)** Magnetic resonance images (MRI) image 3 months post-operatively showing cauda equina damage. Red circles highlight the mild fracture with relatively preserved vertebral shape. **(D)** Posterolateral radiograph 4 months post-operatively, showing a crater-like osteoporotic lesion (red circle) in both L2 and L3, with more severe involvement in L2. **(E)** Sagittal (left) and transverse (right) CT images showing vacuum clefts on L2 and L3 (red circles), an osteophyte protruding into the spinal canal, and L2 and L3 fractures. **(F)** MRI showing spinal cord compression (red circle), with the severity evident in the transverse section (red rectangle).

At 3 months post-operatively, the patient presented with recurrent lower back pain persisting for 1 month and a visual analogue score of 2 out of 10. The vertebrae maintained a rectangular shape on lateral views, and no vertebral body compression was observed (Figures 2A,B). Magnetic resonance imaging revealed L3 vertebral fractures secondary to screw removal, however, computed tomography revealed preserved overall vertebral morphology (Figure 2C). Consequently, conservative treatment was initiated, and the patient was monitored closely. Challenge occurred 4 months post-removal, as the patient's symptoms worsened and were characterized by the onset of neurological deficits. She developed lower limb weakness, which was confirmed through physical examination. Muscle tone was increased, and posterior superficial sensation had regressed. Neurological examination revealed bilateral hip flexion and knee extension strengths of 3/5, right ankle dorsiflexion of 4/5, left ankle dorsiflexion of 2/5, left hallux dorsiflexion of 2/5, and right hallux dorsiflexion of 3/5. The patient had a hyperactive tendon reflex (++) but exhibited normal pathological reflex signs and peripheral pulses. Her visual analogue scale score was 6 out of 10. Posterolateral radiograph revealed a significant osteoporotic fracture of the L2 vertebrae, characterized by severe compression, along with fractured L3 vertebrae with collapsed cephalic endplates (Figure 2D). Computed tomography scan that indicated an osteophyte protruding deep into the spinal canal, which was confirmed using magnetic resonance imaging, showing spinal cord injury at the fracture site (Figures 2E,F).

Based on a comprehensive evaluation, the patient was diagnosed with L2 and L3 lumbar fractures combined with spinal cord injury.

### 3.2 Results

**Figure 3:**
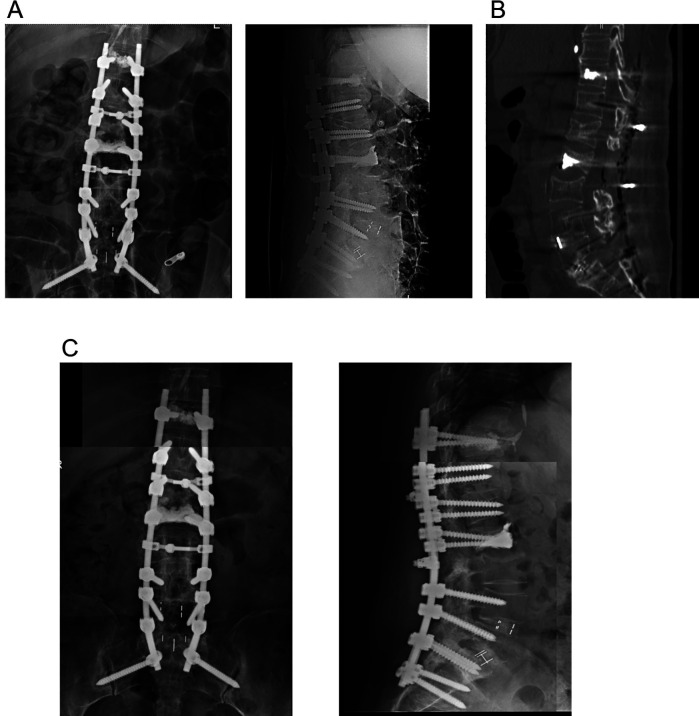
**(A)** Postoperative posterolateral radiograph after the revision surgery, showing the fixation range from the thoracolumbar vertebrae to the ilium. **(B)** Postoperative CT scan after the revision operation, showing L2 vertebroplasty, T11 screw tract augmentation, and achieved fusion at L5-S1. **(C)** Postoperative posterolateral radiograph 6 months postoperatively, taken during scheduled follow-up.

Complex surgical interventions were performed, including L2 total laminectomy (using a posterior approach) with spinal canal and nerve root decompression, L2-L3 intertransverse bone grafting, L2 vertebroplasty, T11-S2 pedicle screw-rod fixation, and T11 screw tract augmentation. Intraoperative findings included an L2 osteoporotic fracture with an intravertebral vacuum cleft sign, accompanied by the posterior displacement of local bone fragments. At the L2 vertebral level, a lamellar bone fragment from the posterior lamina protruded into the spinal canal, causing severe spinal cord compression, evidenced by a visible flattening. In addition, adhesive bands circumferentially compressed the spinal cord. Furthermore, a minimally displaced L3 fracture was observed. Postoperative imaging revealed adequate instrumentation alignment (Figures 3A,B).

At the 6-month follow-up after the third surgery, the patient reported significant symptom relief, with lower extremity strength graded at 4+/5 and a visual analogue scale score of 1/10. Dual-energy x-ray absorption revealed decreased and normal density (*T* = −0.6 in the femur). These findings were further validated using radiography (Figure 3C).

**Figure 4:**
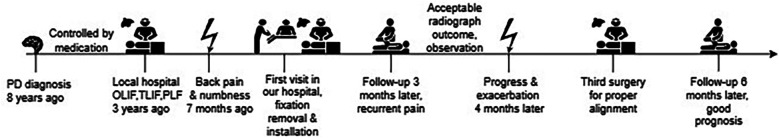
The patient's condition development timeline.

To visualize this process, we drew a scheme graft showcasing the episodes of care (Figure 4).

## 4 Discussion

Pedicle screws are extensively used for internal fixation and bone-graft fusion. Finite element analysis of L3–L4 single-segment posterolateral fusion surgery has revealed that removing pedicle screws can increase mobility at the fused segment, relieving adjacent disc pressure and potentially lowering the incidence of vertebral degeneration (6). However, the question remains whether pedicle screw constructs should be routinely removed after confirmed fracture healing or fusion.

We collectively report five cases from the previous studies in which vertebral body fractures occurred after pedicle screw removal (Table 1).

**Table 1:** Cases of adjacent vertebrae fracture after pedicle screw removal.

Author	Year	sex	Age (years)	Medical history	Bone density (T-score)	Diagnosis	Primary lesion segment	Time to removal	Secondary lesion segment	Recurrence time	Treatment	Prognosis
Waelchli et al. (10)	2002	F	53	PLF	Osteoporosis	Lumbar fracture	L1	18 months	T12	3 days	Orthosis, analgesics	Kyphosis without progression, moderate to severe backpain
Waelchli et al. (10)	2002	F	62	PLF	Osteoporosis	Lumbar fracture	L2	12 months	L1	2 days	Orthosis	Healed fracture, occasional back pain
Cappuccio et al. (7)	2015	M	29	Bronchitis severe coughing	Normal	Lumbar fracture	L2	9 months	L1	1 month	Orthosis, analgesics	Recovered after 3 months of therapy
Kim et al. (8)	2016	F	67	PLF	−1.8	DLS	L2-L3 L3-L4 PLF + L45 PLIF	7 years	L3(T12-L2)	5 weeks	Orthosis, analgesics	Kyphosis and back pain, required the use of a crunch.
Mataki et al. (9)	2018	F	66	Falling	−1.56	DLS	L4-S1 PLIF	10 years	L5(L3-L4)	2 weeks	Surgery, teriparatide	No discomfort, normal ambulation

F, female; M, male; PLF, posterior lumbar interbody fusion; DLS, degenerative lumbar scoliosis; L, lumbar vertebra; S, sacral vertebra; T, thoracic vertebra.

Table 1 reveals that primary risk factors for complications after screw removal include advanced age (60 years), timing of removal [early (7) or late (8, 9)] and osteoporosis (8–10). Notably, advanced age and osteoporosis are commonly observed in patients with PD.

In our case, the patient initially presented a mild fracture and preserved vertebral shape, but subsequently developed a screw tract-related compressive fracture after non-operative treatment. Similar cases were reported by Waelchli et al. (10). However, the fractures occurred much more rapidly in those cases than they did in the present case. Additionally, although vertebral fractures occurred, conservative therapy was effective, as it halted the progression of kyphosis in both patients in their study. In contrast, our patient's fracture occurred later and progressed after hardware removal. Several plausible explanations may account for this difference.

First, non-operative treatment, particularly the use of anti-osteoporotic drugs is crucial after fixation removal. A meta-analysis confirmed that these medications can reduce the incidence of vertebral fractures with *in vivo* animal studies showing that they can promote fracture healing (11). Furthermore, patients with PD are prone to weakened posterior muscle tension bands owing to impaired muscle strength and a higher prevalence of Vitamin D deficiency, which increase their risk of vertebral fractures and osteonecrosis (12). Zhan et al. reported a case of a 67-year-old female with 10-year PD history, and a 3-year history of brain infarction, osteoporosis and T11-L1 internal fixation (13). The authors revealed that osteoporosis and related compressive fractures were also prevalent in patients with PD, and that osteoporosis is strongly associated with fracture risk. Wakita et al. reported a case in which an older man without osteoporosis underwent L2-S1 OLIF, L3-L4, L4-L5 facetectomy, and T10-S1 posterior fusion (14). During the 3 months of follow-up, the only complication observed was transient muscle weakness of the lower extremities. This comparison highlights the significance of healthy bone mineral density in postoperative patients. Given that pedicle screw tunnels do not affect the biochemical properties of the vertebral body (15), we propose that the L2 fracture in our patient was primarily owing to low bone density. As the fixation device reduces mechanical stress on the vertebrae, we recommend retaining them, even after confirmation of healing, until bone density is stable or mildly improved through appropriate treatment after proper treatment.

Second, in patients with PD, decreased daily physical activity may reduce spine mechanical loading, potentially delaying fracture manifestation. However, uncontrolled tremors may exert unpredictable forces, potentially causing progressive adjacent segment disease, as observed in our case. Waelchli et al. reported cases where fractures developed within days of hardware removal (10). In contrast, fractures (in our case and Zhan's cases) occurred months later (13). In a case reported by Cappuccio et al., a sudden increase in spinal load from coughing triggered a secondary vertebral fracture (7). This highlights the significance of evaluating systemic conditions that may increase spinal stress before removing internal fixation devices. Conversely, in Wakita's case, no known trauma or episodic event caused to a direct L4 fracture (14), as observed in our patient. They concluded that in patients with PD, unpredictable muscle tremors and micromovements could affect primitive bone union, particularly in short-segment fixation. This phenomenon may interfere with the early stages of the healing process, causing non-union. This, combined with osteoporosis, may explain why patients with PD are more prone to fractures in the absence of trauma. Controlling tremors and abnormal muscle tension may mitigate fracture risk.

Luo et al. revealed that PD severity is positively correlated with serum vitamin D levels (16). Because increased serum vitamin D levels may promote calcium absorption and outdoor physical activity can further improve muscle strength and endogenous vitamin D synthesis, we encourage patients with PD to engage in supervised outdoor activitie. This could lower the risk of tremor-induced healing failure and stress accumulation, enhance bone mineral density, and decrease the risk of fracture from sudden mechanical load. However, considering the potential risk to patients with osteoporosis, we also recommend deferring fixation device removal until bone density stabilizes or mildly improves after appropriate treatment.

Finally, we recommend that patients with PD retain their fixation devices, as this may reduce complication severity. Kim et al. reported the case of a 67-year-old female with a history of PD and prior L2-L5 fusion who underwent L2-L5 fixation removal, posterior decompression, and additional fusion at T12-L2 suggesting that fixation removal could predispose patients to more severe fractures or spinal deformities (8). This patient's clinical course was similar to that of our patient, with both experiencing progressive adjacent vertebral fractures. Additionally, Kim et al. suggested that although there are “standards” for fixation removal, including local irritation, patients’ preference, and radiographic confirmation of solid fusion, these criteria may not guarantee safety. In our patient, the fixation was removed owing to local nonunion, and a short-range fixation was selected to minimize discomfort; however, an unanticipated fracture occurred. This finding is consistent with that of Kim et al.

Final T12-S2AI fixation was performed in our case. Although minimally invasive spine surgery offers benefits such as reduced muscle trauma and shorter hospital stays in patients with PD-related spinal conditions (17), longer-term follow-up is required to assess its biomechanical stability. Sato et al. reported two cases in which T4-ilium fixation was used for PD-related spinal deformities and suggested that stooped posture typical of PD, cause the spine to act as an extended lever arm under axial load (18). Similarly, Kim et al. speculated that sagittal imbalance contributes to lever-like spinal behavior. This causes a cantilever effect, creating differential stress between the upper and lower spinal column surfaces, increasing fracture risk (8). Sato et al. recommended long-segment fixation to minimize this imbalance, revealing that the thoracolumbar junction, sacrum, and ilium offer significantly greater stability than the lumbar spine does (18). This recommendation was further supported by Hasegawa et al. (19), who used this technique in the treatment of patients with unstable L2-L3 intervertebral space, yielding a favorable prognosis after 1.5 years of follow-up. This technique can increase fixation stability and significantly reduce complications, potentially eliminating the need for removal. However, further studies and long-term observations are required to validate this approach.

This study has some limitations. First, as a single case report, findings cannot be generalized. Hence, further studies involving a larger cohort are required for a more universal conclusion. Furthermore, we discussed reasons for vertebral fracture after fixation removal and the necessity of retaining fixation; however, optimal management, recurrence rate, and risk factors for patients with PD warrant further elucidation. Finally, we primarily focused on the second fracture, which appeared as a stable fracture but developed rapidly. The initial fracture and its cause are rarely discussed. We believe this is owing to its similarity with the second fracture. We observed the entire developing process in the second post-surgical fracture, however, only the patient's outcome was noted during the first episode.

Fractures after fixation removal in patients with PD may result from multiple contributing factors, including osteoporosis, external trauma, accumulated tremors, reduced muscle strength, and insufficient lumbar support. Retaining internal fixation devices may reduce the risk and severity of such fractures. Additionally, anti-osteoporotic treatment, routine physical activity, effective symptom control in PD, and adequate vitamin D supplementation are recommended. Whether long-segment fixation provides additional benefits in patients with PD remains unclear and warrants further investigation.

## Data Availability

The original contributions presented in the study are included in the article/Supplementary Material, further inquiries can be directed to the corresponding author.
